# Epi-GTBN: an approach of epistasis mining based on genetic Tabu algorithm and Bayesian network

**DOI:** 10.1186/s12859-019-3022-z

**Published:** 2019-08-28

**Authors:** Yang Guo, Zhiman Zhong, Chen Yang, Jiangfeng Hu, Yaling Jiang, Zizhen Liang, Hui Gao, Jianxiao Liu

**Affiliations:** Hubei Key Laboratory of Agricultural Bioinformatics, College of Informatics, Huazhong Agricultural University, Wuhan, 430070 People’s Republic of China

**Keywords:** Epistasis, Genetic algorithm, Tabu, Bayesian network

## Abstract

**Background:**

Mining epistatic loci which affects specific phenotypic traits is an important research issue in the field of biology. Bayesian network (BN) is a graphical model which can express the relationship between genetic loci and phenotype. Until now, it has been widely used into epistasis mining in many research work. However, this method has two disadvantages: low learning efficiency and easy to fall into local optimum. Genetic algorithm has the excellence of rapid global search and avoiding falling into local optimum. It is scalable and easy to integrate with other algorithms. This work proposes an epistasis mining approach based on genetic tabu algorithm and Bayesian network (*Epi-GTBN*). It uses genetic algorithm into the heuristic search strategy of Bayesian network. The individual structure can be evolved through the genetic operations of selection, crossover and mutation. It can help to find the optimal network structure, and then further to mine the epistasis loci effectively. In order to enhance the diversity of the population and obtain a more effective global optimal solution, we use the tabu search strategy into the operations of crossover and mutation in genetic algorithm. It can help to accelerate the convergence of the algorithm.

**Results:**

We compared *Epi-GTBN* with other recent algorithms using both simulated and real datasets. The experimental results demonstrate that our method has much better epistasis detection accuracy in the case of not affecting the efficiency for different datasets.

**Conclusions:**

The presented methodology (*Epi-GTBN*) is an effective method for epistasis detection, and it can be seen as an interesting addition to the arsenal used in complex traits analyses.

**Electronic supplementary material:**

The online version of this article (10.1186/s12859-019-3022-z) contains supplementary material, which is available to authorized users.

## Background

With the rapid development of many high-throughput technologies, massive biological data has been produced in recent years, such as genome, transcription and phenotype data. It is possible to mine genetic loci affecting specific phenotypic traits (such as agronomic traits, human diseases, etc.) using the genome data. And it has also become a challenging research topic in today’s biological field. Genome-Wide Association Study (GWAS) is a common method for detecting Single Nucleotide Polymorphism (SNP) associated with phenotypes in the whole genome. This method mainly focuses on the detection of major genes, but it cannot detect gene-gene interactions, or epistasis, mainly embodied in the interaction between SNPs. It needs to develop new approaches to mine the epistatic interactions for specific phenotypic traits.

At present, the following four kinds of methods are mainly used for the epistasis detection in case-control study: statistical method, information entropy method, multi-stage method, machine learning method.

(i). Statistical method. The logistic regression is a method used earlier to detect epistasis [1]. Later the improved logistic regression based on Group Lasso method is used into epistasis mining [2]. The result of logistic regression method is easy to explain, but it has the problems of overfitting, model decline and large amount of calculation. Other statistical methods mainly include functional regression model [3], statistical epistasis networks [4], variance analysis-based method named FastANOVA [5], etc. However, the efficiency of statistical methods is often not high and it needs to set more statistical factors and more complex parameters. Multifactor-Dimensionality Reduction (MDR) is a commonly used epistasis detection approach. But it adopts the exhaustive search strategy, leading to large amount of computation [6]. Subsequently, some researchers improved the MDR method, including MB-MDR, GMDR, FMDR, QMDR, UM-MDR [7], Crush-MDR, KNN-MDR, CMDR [8] and so on. In all, the MDR related methods are more complex when to deal with multiple locus, and the results obtained by these methods are difficult to explain, resulting in poor practicability. Bayesian theory is also used for epistasis mining. The representative method is Zhang’s BEAM method [9], and subsequent improvement methods, including BEAM2, BEAM3 and JBASE [10]. However, the Bayesian theory related methods are complex, inefficient and with insufficient accuracy.
(ii). Information entropy method. In 2008, Dong et al. applied the information entropy theory to detect gene loci epistasis, and verified this method on simulated and real malaria dataset [11]. Hu et al. used the information gain approach to detect three-way epistatic interactions [12]. Kwon et al. dealt with the low-order and high-order epistatic interaction respectively based on the information entropy theory [13]. Li judged the epistasis using information entropy and Bayesian network K2 scoring method [14]. Besides, some research work use the information gain method, such as MBS-IGain [15], Exhaustive-IGain [16].
(iii). Multi-stage method. This kind of method firstly screens out a few important loci and then detects epistasis of the selected important locus. The representative methods include *SNPHarvester* [17], *SNPRuler* [18], *LEAP* [19], *EPIQ* [20], etc. On the basis of Boolean operation, *BOOST* detects epistasis using the stages of screening and testing [21]. The efficiency of this method is relatively high, but it is limited to the interaction between two SNPs, which leads to limited utility. The most important step in multi-stage method is to find the appropriate screening criteria, but it is easy to leave out some important epistasis loci.
(iv). Machine learning method. At present, machine learning methods are increasingly used for mining epistatic loci, such as random forest [22], support vector machine [23], association rules [24], neural network, etc. However, the biggest drawback of machine learning method is that the result is difficult to explain, and tends to overfitting. It often requires cross validation, resulting in high computation cost. In order to improve the detection efficiency and get the global optimal solution, some researchers use the evolutionary algorithm into epistasis mining, such as genetic algorithm (GA) [25], particle swarm optimization [26], etc. Ant colony algorithm is a frequently used heuristic search method for epistasis detection, including *AntEpiSeeker* [27], *AntMiner*, *MACOED* [28], *epiACO* [29], *FAACOSE* [30], etc. However, ant colony algorithm has some disadvantages, such as difficulty in determining the control parameters, premature stagnation and slow convergence in the early stage, which will affect the calculation accuracy.

Compared with other methods, Bayesian network has the advantages of constructing the causal relationship between objects, mining implicit knowledge, processing data with nonlinear relationship and noise, dealing with different data types, etc. In recent years, some research work use Bayesian network learning method to construct the network of gene loci and phenotype, and thus to detect the epistatic loci for specific phenotype [31, 32]. In this work, we firstly construct the network of gene loci for specific phenotype using Bayesian network, and then mine the epistasis for specific phenotype. However, due to Bayesian network usually uses the partial or random search strategy, it is easy to fall into local optimum and further to influence the learning accuracy. Genetic algorithm has the characteristic of rapid global search and avoiding falling into local optimization. In this approach, we use the genetic algorithm into the heuristic search strategy of Bayesian network. The evolution of individual structure is realized through three genetic operations (selection, crossover, mutation), and thus to find the optimal network structure. Inspired by the genetic tabu algorithm used in [33, 34], we use the tabu search strategy into the crossover and mutation operation of genetic algorithm. It can help to enhance the diversity of population and thus to obtain the global optimal solution. In the genetic algorithm, the quality of the initial population has an important effect on the result. We use mutual information entropy calculation method to calculate the relationship between gene loci and phenotype, and thus to construct the initial network. It can help to enhance the quality of the initial network. In order to speed up the calculation, we convert the genotypic data into binary Boolean data and then directly carry out the fast logic (bitwise) operation to calculate the mutual information. The simulated and real datasets are used to validate the proposed *Epi-GTBN*, and we compare it with other recent algorithms. Experiment results show *Epi-GTBN* has much better epistasis detection accuracy in the case of not affecting the efficiency.

## Results

The *bnlearn* [35] is an *R* package for learning the graphical structure of Bayesian network, estimating their parameters and performing some useful inference (http://www.bnlearn.com/). The source code of this package is open, so we can modify and compile the source code conveniently. This package implements several kinds of constraint-based, score-based and *hybrid* structure learning algorithms, such as *IAMB*, *mmpc*, *hc*, *mmhc*, etc. On the basis of the source code of *bnlearn* package, we have implement the proposed *Epi-GTBN* using *R*, see http://122.205.95.139/Epi-GTBN/.

### Experiments on simulated data

The experiment is carried out on the computer with the configuration of Intel(R) Core (TM) i7–4790 CPU@ 3.60GHz 4.00GHz, and 8G memory. GAMETES is a commonly used software for the epistasis data generation [36]. It can generate epistasis simulated data quickly and accurately, and generate two or multi-locus epistasis models by setting different parameters. The parameters that can be set in this software include: number of SNP loci, heritability, minimum allele frequency (MAF) and prevalence. Heritability is a measure of how well differences in genes account for differences in the traits. A heritability close to zero indicates that almost all of the variability in a trait is due to environmental factors, with very little influence from genetic differences. MAF refers to the frequency of unusual alleles in a given population. In the simulation file, the last column is phenotype *Class*, 1 represents case, 0 represents control. It uses 0, 1, 2 to express the genotype data, 0 denotes homozygote common genotype, 1 denotes heterozygous genotype and 2 denotes homozygote rare genotype.

Through setting different values of heritability *h*^*2*^ and MAF, we use GAMETES to generate different simulated datasets. Each dataset includes 100 files. To evaluate the performance of the epistasis detection algorithm, we use Eq.(1) to calculate the detection accuracy.

1 $$ Accuracy=\frac{Num_{edge}}{100} $$

In the equation, *Num*_*edge*_ refers to the number of datasets in which the disease associated SNPs are successfully identified among all 100 datasets generated by the same parameters.

Among existing approaches, *BEAM* [9], *AntEpiSeeker* [27], *SNPRuler* [18], *MDR* [6], *BOOST* [21] are powerful tools for the detection of epistatic interactions. In order to do the validation, we compare our *Epi-GTBN* with these tools on the simulated datasets. In addition, we use the frequently-used Bayesian network learning algorithm of *hill-climbing* (*hc*) to do comparison. In this section, we do the detection accuracy and efficiency comparison regarding 2-locus and 3-locus epistatic interaction with heritability *h*^*2*^ set to 0.025, 0.05, 0.1, 0.2, 0.3, 0.4 and MAF set to 0.1, 0.2, 0.3, 0.4.

Generally, population size is set to 50–100 and crossover probability is set about 0.6 [37]. Mutation probability is generally set as 0.005–0.01 [38]. The tabu list length can’t be set too long or too short, such as more than or far less than the population size. The GA algorithm always converges within 60 iterations in our previous experiment. In our *Epi-GTBN*, we set the population size to 50, set crossover probability to 0.7, set mutation probability to 0.002, set the length of tabu list in the crossover operation to 30, set the maximum number of iterations to 60, set the generations of *k* to 3 when fitness value of the optimal individual and the population no longer increases.

There are no parameters used in *BEAM*, *MDR* and *BOOST*. In *SNPRuler*, we set *listSize* to 2000, *depth* to 4 and set *updateRatio* to 0.5. These parameters have no effect on the result in our previous experiments. Population size is the only same parameter in both *AntEpiSeeker* and *Epi-GTBN*. So in *AntEpiSeeker*, we also set population size to 50 to ensure the fairness of the experiment. Similarly, we set the maximum number of iterations to 60 in *hill-climbing*.

#### Experiment of 2-locus epistasis detection

**Fig. 1:**
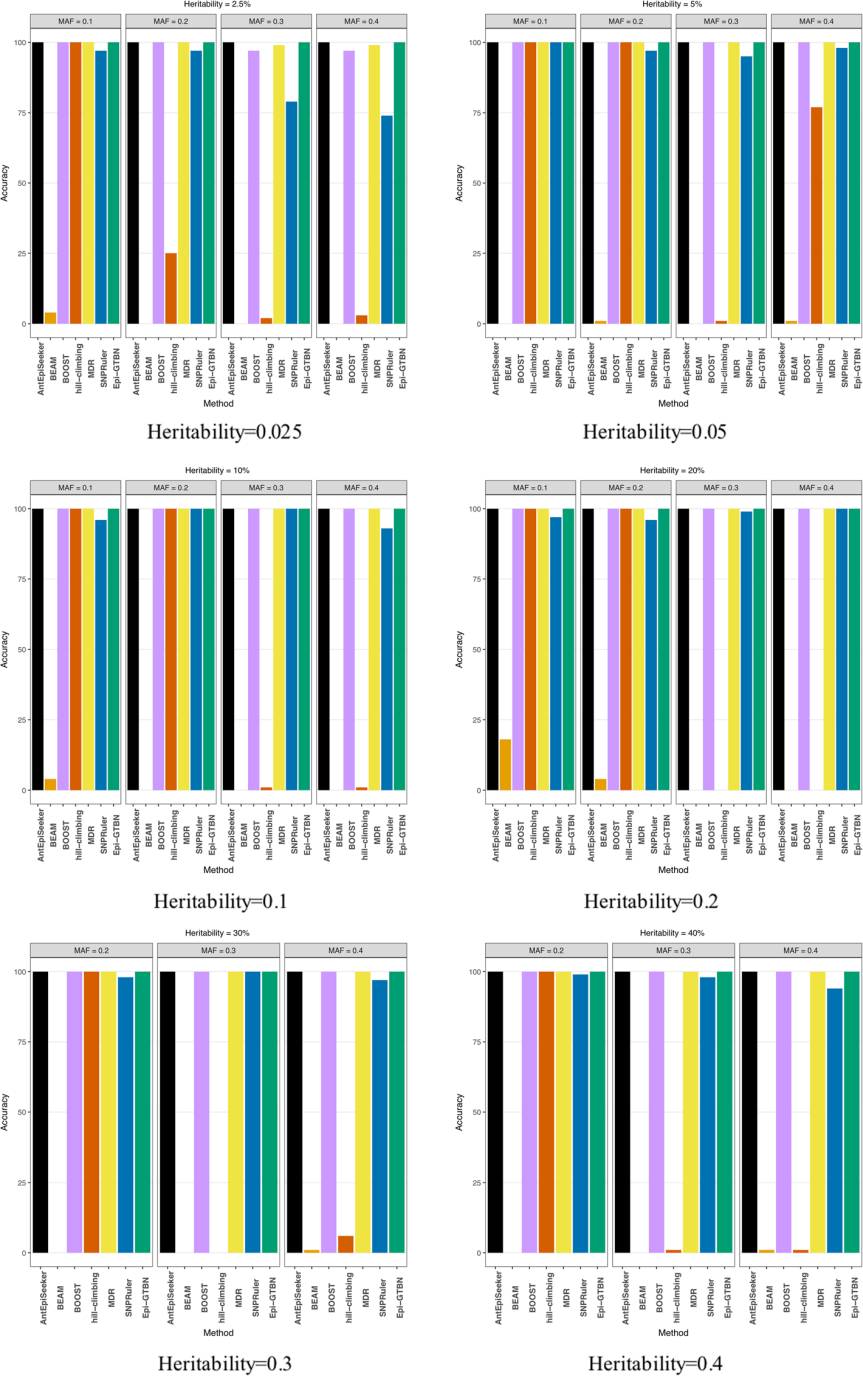
2 locus epistasis detection accuracy comparison of different methods

**Fig. 2:**
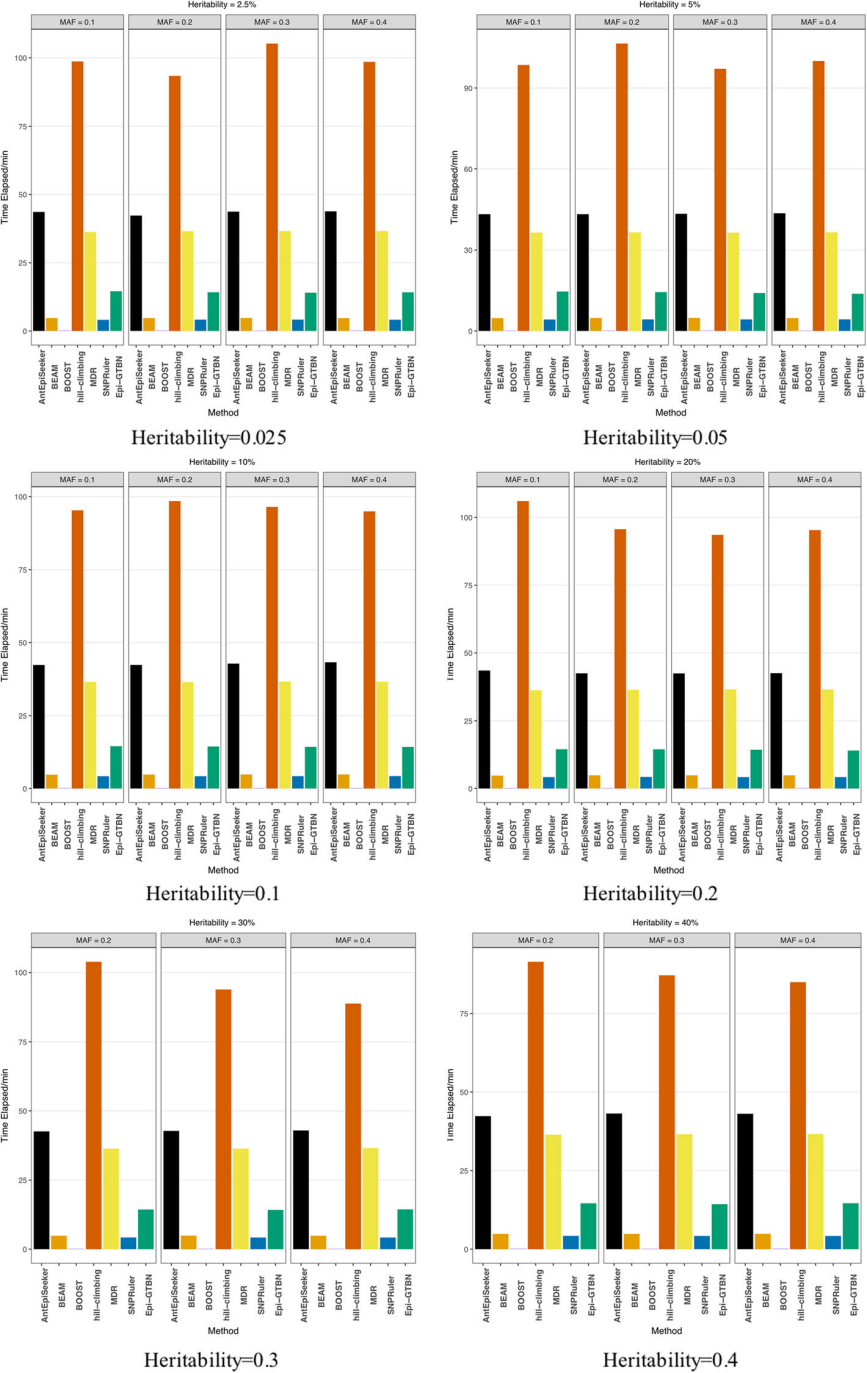
2 locus epistasis detection efficiency comparison of different methods

In this experiment, we compare the detection accuracy of 2-locus epistasis mining in the case of setting different heritability and MAF. Figure 1 and Fig. 2 show the detection accuracy and efficiency comparison of different methods (*AntEpiSeeker*, *BEAM*, *BOOST*, *hill-climbing*, *MDR*, *SNPRuler* and *Epi-GTBN*).

In Fig. 1, in the case of setting different heritability and MAF, we can see the 2-locus epistasis detection accuracy of *BEAM* and *hill-climbing* (*hc*) Bayesian network learning method is far less than other 4 kinds of methods. The accuracy of *Epi-GTBN*, *MDR*, *BOOST* and *AntEpiSeeker* is the largest of all, mostly hold steady at 100%. The accuracy of *SNPRuler* is slightly less than the above 4 kinds of approaches.

In Fig. 2, we can see the epistasis detection time of *hill-climbing* is the most of all, and it is far larger than the other 6 kinds of methods. The detection time of *BEAM*, *BOOST* and *SNPRuler* is the least of all, and the using time of *AntEpiSeeker*, *MDR* and *Epi-GTBN* is in the middle. The detection time of *Epi-GTBN* is less than *AntEpiSeeker* and *MDR*. In our *Epi-GTBN* approach, we convert the genotypic data into binary Boolean data, and use the fast logic (bitwise) operation directly to calculate the mutual information. This can save a lot of time of calculating the mutual information entropy between any two SNPs and *Class* when to construct the initial network.

In all, the detection accuracy of *MDR*, *BOOST* and *AntEpiSeeker* is same as our *Epi-GTBN* method, mostly hold steady at 100%. But the detection efficiency of *AntEpiSeeker* and *MDR* is lower than *Epi-GTBN* apparently. In addition, the parameter setting of *AntEpiSeeker* is complicated, and its result is related to parameters setting. *BOOST* can only detect the 2-locus epistasis, and it can’t be used for the multi-locus epistasis detection. From the experiment results, we can see the epistatic detection approach based on genetic tabu algorithm and Bayesian network (*Epi-GTBN*) has much better detection accuracy in the case of not affecting the efficiency.

#### Experiment of 3-locus epistasis detection

**Fig. 3:**
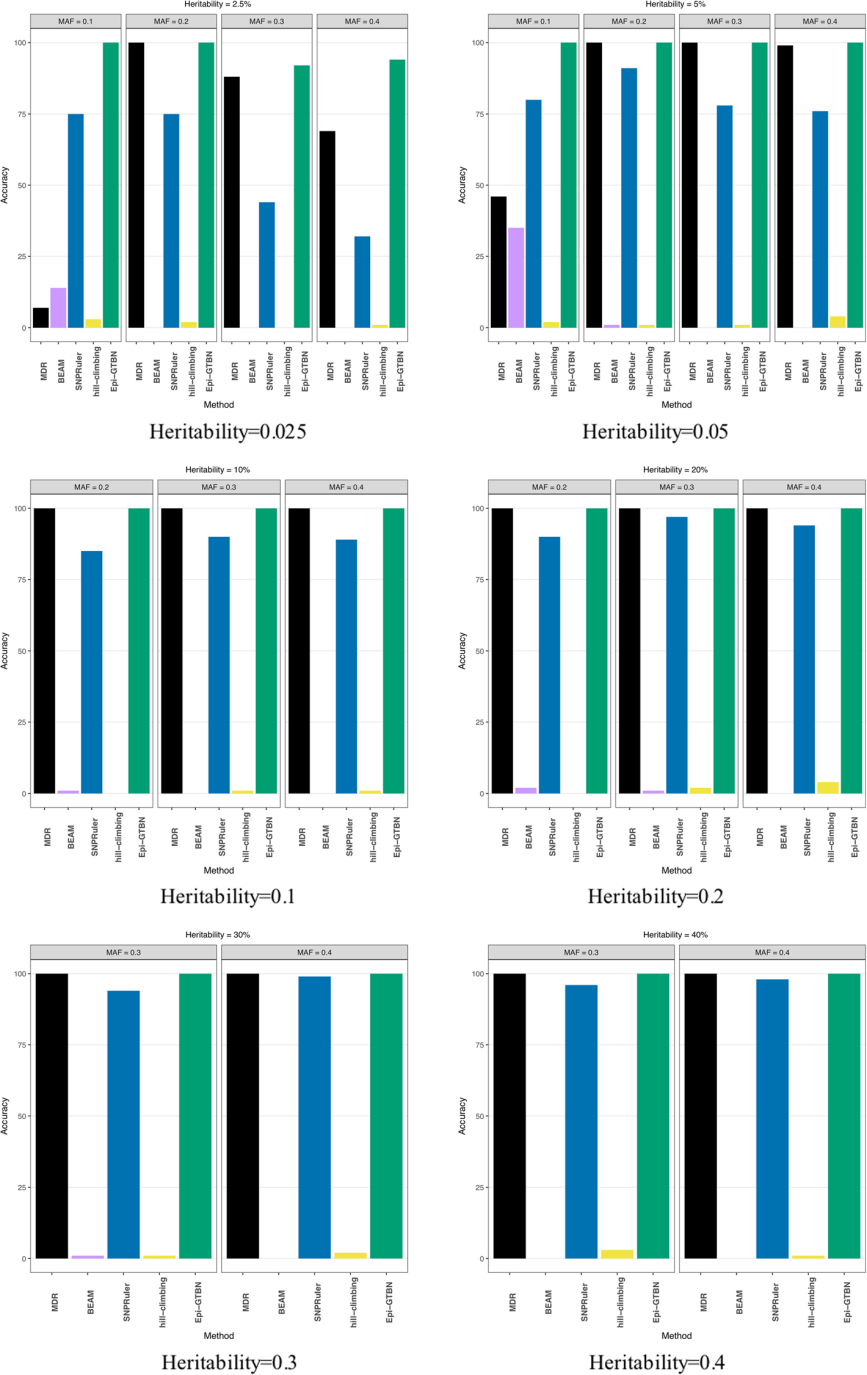
3 locus epistasis learning accuracy comparison of different methods

In this experiment, we compare the accuracy of 3-locus epistasis mining in the case of setting different heritability and MAF. Figure 3 illustrates the detection accuracy comparison of different methods (*MDR*, *BEAM*, *SNPRuler*, *hill-climbing* and *Epi-GTBN*).

The 3-locus epistasis detection accuracy shown in Fig. 3 is almost the same as the case of 2-locus epistasis detection illustrated in Fig. 1. The detection accuracy of *BEAM* and *hill-climbing* (*hc*) method is the least of all. The accuracy of *Epi-GTBN* and *MDR* is the largest of all, at around 100%, which is slightly more than *SNPRuler*.

### Experiments on real AMD data

In order to prove the effectiveness of *Epi-GTBN*, we also use the real age-related macular degeneration (AMD) dataset, which contains 103,611 SNPs genotyped with 96 cases and 50 controls [39]. AMD refers to pathological changes in the central area of the retina, and it is the most important cause of irreversible visual loss in elderly populations. AMD is considered as a complex disease whereby multiple SNP-SNP interactions interact with environmental factors to it. The AMD dataset has been widely used as a benchmark dataset to validate the epistasis mining approaches [13, 26, 29, 40–44].

After the filtration, there are 102,926 autosomal SNPs remained. Firstly, SNP loci with *p*-values from Chi square test less than 0.01 are removed from AMD dataset. Subsequently, 1039 SNP loci remain in the AMD dataset. Here, we explore 2-SNP AMD-associated genetic factors to validate the effectiveness of *Epi-GTBN*. We calculate the conditional mutual information between any two SNPs and phenotype firstly. Then we sort the SNP pairs and extract the SNP pairs whose mutual information is larger than 0.16. These SNP pairs are used to construct the initial network, and then *Epi-GTBN* is used to learn the Bayesian network of SNP loci and phenotype. There are 171 SNP-pairs in the final output set (see epistatic interactions of AMD.xlsx in Additional file 1). Table 1 shows the Top-10 epistatic interactions that *Epi-GTBN* have detected, which we compared with the results of other four methods that showed promising results when experimenting with simulated datasets (*AntEpiSeeker*, *MDR*, *BOOST*, *SNPRuler*). In the table, the column of *MI* means the calculated mutual information *I* (*Class* | *SNP*_*1*_, *SNP*_*2*_) of *SNP*_*1*_ and *SNP*_*2*_ in the initial network. If a SNP-pair detected by *Epi-GTBN* was also detected by other method, it will be marked in the table. And if that SNP-pair also happens to be one of the top gene-gene interactions detected by other methods, additional information will be added into related cell in parentheses. We have put the full result of Top-10 epistatic interactions captured by *AntEpiSeeker*, *SNPRuler*, *BOOST*, *MDR* in Table 2 for reference.

**Table 1:** Top-10 epistatic interactions associated with AMD captured by Epi-GTBN compare with other methods

ID	SNP 1	SNP 2	MI	References	AntEpiSeeker	MDR	BOOST	SNPRuler
1	rs380390	rs1363688	0.205025859	Sun et al. 2017, Shang et al. 2014, Tuo et al. 2016, Shang et al. 2015	–	✓ (11)	–	–
2	rs380390	rs2402053	0.204420493	Sun et al. 2017, Tuo et al. 2016, Shang et al. 2015 Han et al. 2012	–	–	–	–
3	rs380390	rs10512174	0.192477486	Sun et al. 2017, Shang et al. 2015	–	–	–	–
4	rs380390	rs718263	0.192360092	Sun et al. 2017, Shang et al. 2015	–	–	–	–
5	rs1329428	rs9328536	0.190001652	Sun et al. 2017, Kwon et al. 2014, Tuo et al. 2016	✓ (top-10)	–	–	–
6	rs1329428	rs7467596	0.190001652	Tuo et al. 2016	–	–	–	–
7	rs10503216	rs9316435	0.188192429	–	✓ (top-10)	✓	✓	–
8	rs380390	rs335368	0.184951682	–	–	–	–	–
9	rs380390	rs555174	0.184735375	–	–	✓ (top-10)	–	–
10	rs380390	rs724972	0.183950563	Tuo et al. 2016	–	✓ (top-10)	–	–

**Table 2:** Top-10 epistatic interactions associated with AMD captured by AntEpiSeeker, SNPRuler, BOOST, MDR

ID/Methods	AntEpiSeeker		SNPRuler		BOOST		MDR	
	SNP1	SNP2	SNP1	SNP2	SNP1	SNP2	SNP1	SNP2
1	rs1329428	rs9328536	rs10503790	rs6928748	rs9316435	rs10503216	rs555174	rs380390
References	Sun et al. 2017, Kwon et al. 2014, Tuo et al. 2016		–		–		–	
2	rs4880042	rs718309	rs657618	rs7908635	–	–	rs10507949	rs10511467
References	–		–		–		–	
3	rs9316435	rs10503216	rs10512781	rs10510099	–	–	rs1293449	rs380390
References	–		–		–		–	
4	rs10505112	rs10512174	rs215389	rs903645	–	–	rs961360	rs380390
References	–		–		–		–	
5	rs1359634	rs1740752	rs4526387	rs2105250	–	–	rs10511467	rs1394608
References	–		–		–		–	
6	rs1535891	rs6598991	rs485412	rs10497257	–	–	rs724972	rs380390
References	–		–		–		Tuo et al. 2016	
7	rs9294603	rs6540592	rs1677189	rs4947673	–	–	rs261796	rs380390
References	–		–		–		–	
8	rs943653	rs4128956	rs3829918	rs727200	–	–	rs1510134	rs380390
References	–		–		–		–	
9	rs1233255	rs860309	rs7533063	rs10484087	–	–	rs1742923	rs380390
References	–		–		–		–	
10	rs404199	rs10510895	rs1489402	rs10484087	–	–	rs1146382	rs380390
References	–		–		–		–	

**Fig. 4:**
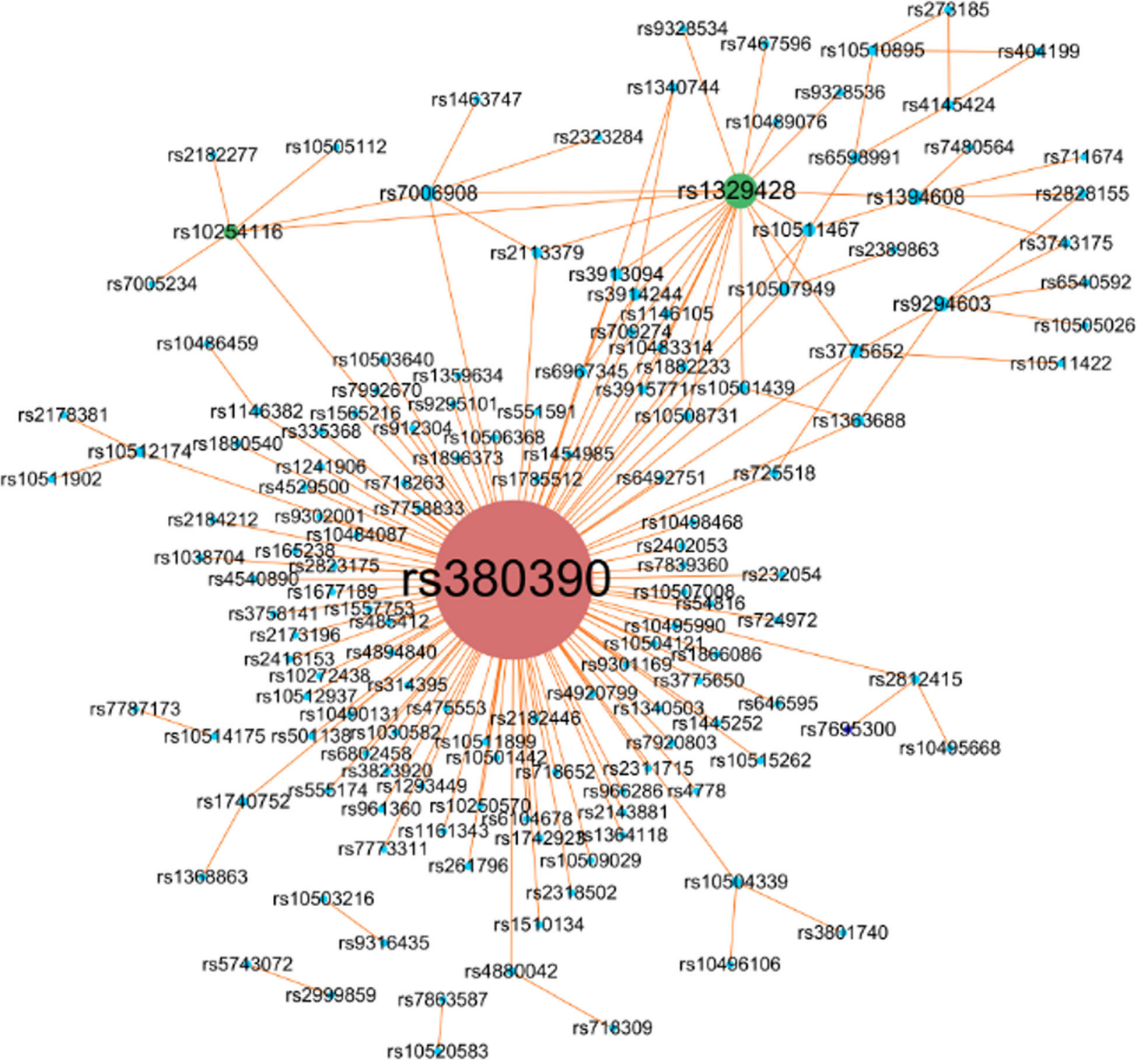
SNP-SNP network of AMD

As we know, how to evaluate the detection accuracy of the algorithms with real data is more difficult compared with the simulated data. This is due to the precise identification of all epistasis for the real data is not known. Therefore, we validate our method by searching for literature support in this work. In Table 1, we can see the Top-10 epistatic interactions detected using *Epi-GTBN* have strong literature support. The interaction (rs380390, rs1363688) and (rs380390, rs2402053) are the most statistically significant two among all detected SNP-SNP interactions, and it has also been reported by many literatures [26, 29, 40, 41, 44]. Similarly, the SNP-SNP interactions (rs380390, rs10512174), (rs380390, rs718263), (rs1329428, rs9328536), (rs1329428, rs7467596), (rs380390, rs724972) have been reported by many literatures. Additionally, besides Top-10 SNP-pairs, seven other SNP-pairs detected by *Epi-GTBN*: (rs380390, rs10512937), (rs380390, rs10483314), (rs380390, rs10507949), (rs1394608, rs3743175), (rs1394608, rs2828155), (rs1329428, rs3775652), (rs3775652, rs725518) received literature supports, as shown in Table 3. All these 171 SNP-pairs are displayed in Fig. 4.

**Table 3:** Other epistatic interactions associated with AMD captured by Epi-GTBN with literature support

ID	SNP 1	SNP 2	MI	References
1	rs380390	rs10507949	0.183066189	Shang et al. 2015
2	rs380390	rs10512937	0.176409436	Tuo et al. 2016
3	rs380390	rs10483314	0.172425422	Tuo et al. 2016
4	rs3775652	rs725518	0.170306079	Tuo et al. 2016
5	rs1329428	rs3775652	0.168639751	Tuo et al. 2016
6	rs1394608	rs3743175	0.162643832	Tang et al. 2009, Jiang et al. 2009
7	rs1394608	rs2828155	0.162643832	Tang et al. 2009, Jiang et al. 2009

In Fig. 4, a node denotes a SNP locus. Two linked nodes represent one SNP-pair of final 171 SNP-pairs. The larger of the node, the more nodes linked with it. It can be seen evidently from Fig. 4 that three SNPs ‘rs380390’, ‘rs1329428’ and ‘rs10254116’ are associated with more other SNPs. This finding is consistent with the work that firstly introduced about the AMD dataset [39], in which the authors reported that two SNPs, ‘rs380390’ and ‘rs1329428’, were believed to be particularly associated with AMD. We hope that, from these results, some clues could be provided for the exploration of causative factors of AMD.

## Conclusion

GWAS focuses on single locus and main effect gene locus detection. Although a lot of phenotype related loci can be found using this method, these loci can only explain very few genetic variations. One of the important reasons is that it does not consider the gene-gene interactions, namely epistasis. Therefore, the detection of epistasis is of great significance to the discovery, diagnosis, treatment and prevention of complex diseases. In this study, we propose an epistasis detection approach called *Epi-GTBN*. The genetic tabu approach is used into the search strategy of Bayesian network, and BIC scoring is used to calculate the fitness function value. *Epi-GTBN* can learn the optimal network structure quickly and accurately, and then used to mine epistatic loci. *Epi-GTBN* has 3 major features: *i*) The mutual information entropy is used in the generation of initial individuals to improve the quality of the initial population, which helps to improve the accuracy of epistasis detection. In order to speed up the calculation, it converts the genotypic data into binary Boolean data, and carries out the fast logic (bitwise) operation directly to calculate the mutual information. *ii*) It combines genetic algorithm with Bayesian network to construct the network of gene loci for specific phenotype. The operations of selection, crossover, mutation are used into the search strategy of Bayesian network, thus to evolve the individual structure to achieve the global optimization. *iii*) The tabu search strategy is applied to the operations of crossover and mutation of genetic algorithm, which enhances the diversity of population, and helps to get the global optimal solution and accelerate the convergence of the algorithm. Experimental results in simulated and real datasets elucidate that *Epi-GTBN* is an effective method for epistasis detection. Compared with other existing approaches, *Epi-GTBN* can detect epistatic loci accurately in the case of guaranteeing efficiency.

The next research work mainly includes the following aspects: the time used in the algorithm increases exponentially when to detect multi-locus epistasis. It needs to combine with other algorithms to perform optimization. In addition, prior knowledge should be borrowed to accelerate the process of search.

## Methods

### Bayesian network

Bayesian network (BN) is a graphical model used to represent the probability distribution among variables. A Bayesian network consists of a directed acyclic graph and a series of conditional probability tables. The directed acyclic graph is used to express the conditional dependence relationships. The conditional probability distribution is used to parameterize the nodes. BN provides a way of expressing causality between variables.

Supposing *X* = {*X*_1_, *X*_2_, …, *X*_*n*_}, *BN* = {*G*, *P*}. *G* = {*V*, *E*}, *G* is a directed acyclic graph of *X*. *V* represents the node set in *G* and each node represents a variable in *X*. *E* represents the edge set in *G*. Each directed edge represents the conditional dependence relationship between the corresponding nodes. If there exists a directed edge from *X*_*j*_ to *X*_*i*_, we call *X*_*j*_ the parent node of *X*_*i*_, and *X*_*i*_ the sub node of *X*_*j*_. *P* represents the conditional probability set of *BN*. *P* = {*P*(*X*_*i*_ | *pa*(*X*_*i*_))}, *P*(*X*_*i*_ | *pa*(*X*_*i*_)) denotes the conditional probability of *X*_*i*_ and *pa* (*X*_*i*_) denotes the parent node set of *X*_*i*_. The full probability distribution of Bayesian network is shown in Eq. (2)

2 $$ P\left({X}_1,{X}_2,\dots, {X}_n\right)=\prod \limits_{i=1}^nP\left({X}_i| pa\left({X}_i\right)\right) $$

Bayesian network structure learning intends to find the optimal network to match the specific dataset. There are mainly two kinds of Bayesian network structure learning methods: the score-based structure learning methods and constraint-based structure learning methods. The score-based structure learning method firstly defines the scoring function, then it uses specific search strategy to find the network structure with the highest score. This method aims to find a graph with the highest fitting degree of the sample data. Due to the huge search space, it often needs a good search strategy to speed up the search process.

### Genetic Tabu algorithm

According to the principle of natural selection, the genetic algorithm (GA) selects the chromosomes that are more suitable for the environment to reproduce. Then it produces a new generation of chromosomes that are more suitable for the environment through the process of crossover and mutation. In this way, it evolves generation by generation, and finally converges to one of the most adaptable chromosomes, so as to find the optimal solution. The genetic algorithm mainly uses three kinds of operations (selection, crossover, mutation) to evolve the population structure, and thus to search for the optimal solution. However, in the searching process of genetic algorithm, it is easy to generate same individual, which affects the diversity of the population. The genetic algorithm selects the better individual and inherits it directly, which is easy to produce local optimal solution, and not conducive to global search.

Tabu search is a famous heuristic search algorithm, which uses the memory function of tabu list to avoid generating some identical individuals, thereby increasing the diversity of population. In addition, tabu search method can accept the inferior solution in the search process, and thus has a stronger climbing ability. This enables tabu search to jump out of the local optimal solution and search for other regions in the search process, thus greatly increase the probability of obtaining better or global optimal solutions. In order to solve the above problems of genetic algorithm, we apply the unique memory function of tabu search into the operations in genetic algorithm inspired from [33, 34]. In [34], solution attributes in tabu list are used for the adjustment of mutation probability in genetic algorithm. In our work, we use the tabu search strategy into the improvement of the crossover operator and mutation operator. This method can be used to improve the performance of the algorithm and find the optimal network structure quickly and accurately.

### Epi-GTBN approach

In this work, we construct the network of gene locus for specific phenotype using Bayesian network, and thus to mine the epistasis interactions. The genetic tabu algorithm is used into search strategy of Bayesian network. This approach mainly includes the following steps: initial network construction, initial network population generation, genetic manipulations (selection, crossover, mutation) of the network, getting epistasis interactions.

#### Network coding

**Fig. 5:**
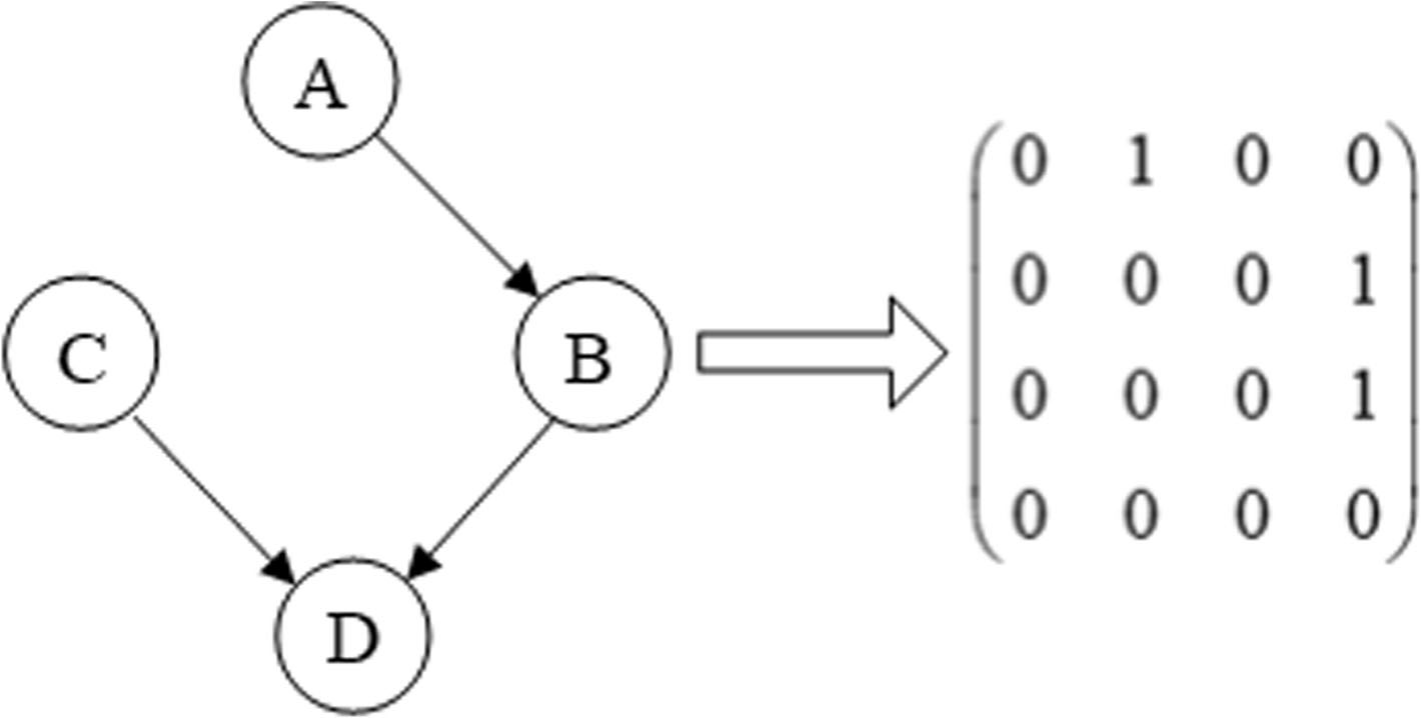
Matrix coding of Bayesian network

In *Epi-GTBN*, each individual in the genetic algorithm corresponds to a Bayesian network structure, and it does the search in the space of Bayesian network structures. We use the adjacency matrix to represent the Bayesian network structure. Supposing the number of variables is *n*, and each individual can be represented as an adjacency matrix *C* of *n* × *n*. We use 0/1 coding approach to represent the matrix. If node *i* is the parent node of node *j*, then *C*_*ij*_ = 1, otherwise, *C*_*ij*_ = 0, as depicted in Fig. 5.

#### Initial network population generation

**Fig. 6:**
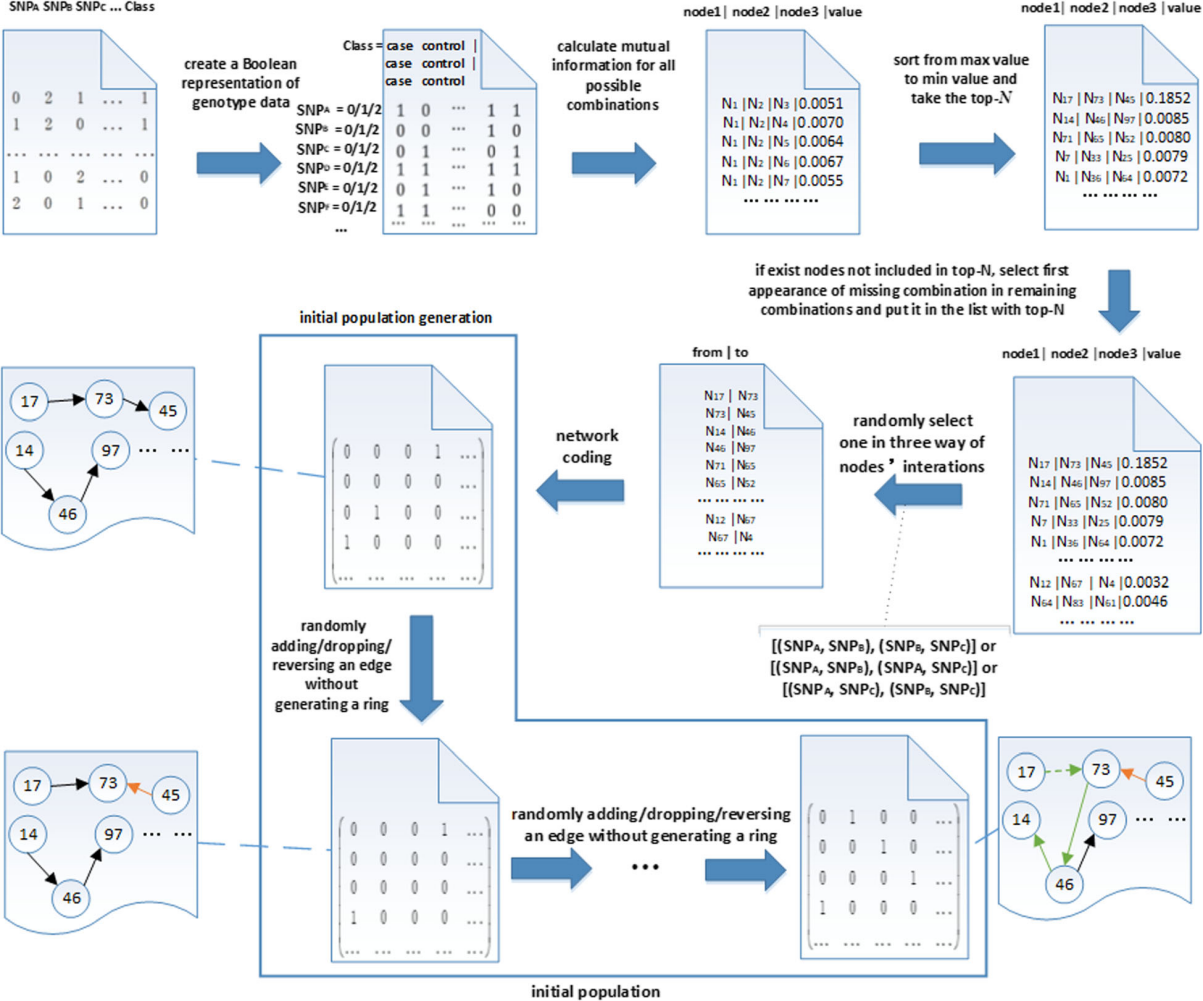
The process of initial population generation

In this work, the initial population refers to a set of different Bayesian network individual. In BN, each node denotes the SNP locus or phenotype, and each edge denotes the association between the nodes of SNP locus or phenotype. The quality of initial population has an important impact on the subsequent network structure learning. We calculate mutual information to express the association between multiple gene locus and phenotype [14], and thus to construct the initial network. In further to enhance the calculation efficiency, we convert the genotypic data into binary Boolean data firstly. Then we can use fast logic (bitwise) operation to calculate the mutual information directly. The concrete process of generating initial population is illustrated in Fig. 6.

In Fig. 6, it firstly converts the genotype data into binary Boolean data, and calculates the mutual information between multiple nodes and phenotype for all the possible combinations. Then it sorts the node pairs according to the value of mutual information and extracts the top-N node pairs. The top-N is determined according to the experiment results. For different datasets, we can extract different number of top-N node pairs. The top-N node pairs may not cover all the nodes, and it means there are nodes that are not included in the top-N node pairs. Then we select the first appearance of these nodes in the remaining node pairs, and also extract the corresponding node pairs. Finally, it constructs the initial network according to the node pairs as the initial individual. It generates next individual through adding an edge, dropping an edge or reversing an edge on the premise of not generating a ring. A new individual is generated on the basis of the next individual, until the number of individuals reaches the population size.

**Fig. 7:**
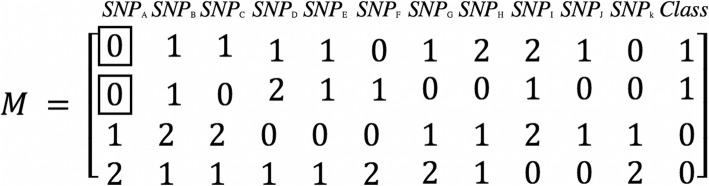
The genotype data

**Fig. 8:**
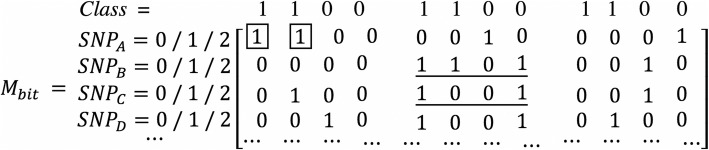
The binary Boolean expression of genotype data

In the first step, we convert the genotype data into binary Boolean format. For example, the genotype data depicted in Fig. 7 is converted into the data format shown in Fig. 8.

In Fig. 7, each column denotes the genotype data of each SNP. In the last column *Class*, 1 denotes the case phenotype and 0 denotes the control phenotype. We can see there are four samples in Fig. 7. In Fig. 8, the first/middle/last four columns denote the binary Boolean expression when the genotype data is 0/1/2 respectively.

We use Eq.(3) to calculate the mutual information between the *k* epistatic SNP loci and *Class* [14]. In Eq.(3), we use Eq.(4) to calculate the information entropy of *Class*, and use Eq.(5) to calculate the joint entropy of *k* SNP locus.

3 $$ I\left( Class|{SNP}_1,\dots {SNP}_k\right)=H(Class)+H\left({SNP}_1,\dots {SNP}_k\right)-H\left( Class,{SNP}_1,\dots {SNP}_k\right) $$

4 $$ H(Class)=-\sum \limits_{ct_i=0}^1p\left({class}^i\right)\log p\left({class}^i\right) $$

5 $$ H\left({SNP}_1,\dots, {SNP}_k\right)=-\sum \limits_{snp_1=0}^2\dots \sum \limits_{snp_k=0}^2p\left({snp}_1,\dots, {snp}_k\right)\log p\left({snp}_1,\dots, {snp}_k\right) $$

On the basis of binary expression of genotype data, we can conduct the logic **AND** operation to calculate the mutual information efficiently. For example, we use Eq.(6) to calculate *I*(*Class* |*SNP*_*B*_, *SNP*_*C*_) shown in *M*_*bit*_.

6 $$ I\left( Class|{SNP}_B,{SNP}_C\right)=H(Class)+H\left({SNP}_B,{SNP}_C\right)-H\left( Class,{SNP}_B,{SNP}_C\right) $$

We use Eq.(7) to calculate *H*(*SNP*_*B*_, *SNP*_*C*_) in Eq.(6).

7 $$ {\displaystyle \begin{array}{l}H\left({SNP}_B,{SNP}_C\right)=-\sum \limits_{snp_B=0}^2\sum \limits_{snp_C=0}^2p\left({snp}_B,{snp}_C\right)\log p\left({snp}_B,{snp}_C\right)=-p\left(0,0\right)\log p\left(0,0\right)\\ {}-p\left(0,1\right)\log p\left(0,1\right)-p\left(0,2\right)\log p\left(0,2\right)-p\left(1,0\right)\log p\left(1,0\right)-p\left(1,1\right)\log p\left(1,1\right)\\ {}-p\left(1,2\right)\log p\left(1,2\right)-p\left(2,0\right)\log p\left(2,0\right)-p\left(2,1\right)\log p\left(2,1\right)-p\left(2,2\right)\log p\left(2,2\right)\end{array}} $$

Using the underlined binary data in *M*_*bit*_, we can calculate *p* (1, 1) using Eq.(8) through the **AND** operation of binary. It can be accomplished by the counting of “1” bits in a bit string (also called hamming weight).

8 $$ {\displaystyle \begin{array}{l}p\left(1,1\right)=\frac{n_{snp_B={snp}_C=1}}{n_{sample}}=\frac{\mathrm{hamming}\ \mathrm{weight}\left[{(1101)}_2{AND}_{bit}{(1001)}_2\right]}{4}\\ {}=\frac{\mathrm{hamming}\ \mathrm{weight}\left[{(1001)}_2\right]}{4}=\frac{2}{4}=0.5\end{array}} $$

#### Selection

The purpose of selection is to select a good individual from the current population so that they have a chance to be the offspring of the next generation. The principle of selection is that the individual with greater adaptability will be selected with larger probability, which embodies the survival of the fittest principle. This work mainly uses the roulette selection method. Supposing the fitness value is *f*_*i*_ about chromosome *i*, then the probability *P*_*i*_ of chromosome *i* being selected is calculated using Eq.(9). In the equation, *num* represents the size of population.

9 $$ {P}_i={f}_i/\sum \limits_{i=1}^{num}{f}_i $$

#### Crossover

Crossover is the most important operation in the genetic algorithm. It can get better individuals in the new generation through the crossover operation, and the new individuals inherit the characteristics of their parents. The commonly used crossover operations include single column crossover, double or multi column crossover, uniform crossover, etc. The multi column crossover refers to a variation of several columns. In order to speed up the convergence rate, we intend to use the multi column crossover method.

Supposing two individuals of *Individual*_*1*_ and *Individual*_*2*_ in the population, it randomly selects two columns *f*_1_, *f*_2_ of *Individual*_*1*_ and *s*_*1*_, *s*_*2*_ of *Individual*_*2*_. The column *f*_*1*_ of *Individual*_*1*_ is exchanged with column *s*_*1*_ of *Individual*_*2*_, and column *f*_*2*_ of *Individual*_*1*_ is exchanged with column *s*_*2*_ of *Individual*_*2*_. *Individual*_*1*_[...*f*_*1*_...*f*_*2*_...] $\overset{\mathrm{crossover}}{\leftrightarrow }$ *Individual*_*2*_[...*s*_*1*_...*s*_*2*_...]. Then we can get *Individual*_*1*_[...*s*_*1*_...*s*_*2*_...] and *Individual*_*2*_[...*f*_*1*_...*f*_*2*_...]. It will also judge whether the crossover operation will generate a ring or not. When there is no ring structure in both *Individual*_*1*_ and *Individual*_*2*_, they will be considered as new offspring.

##### Avoid ring generation

**Fig. 9:**
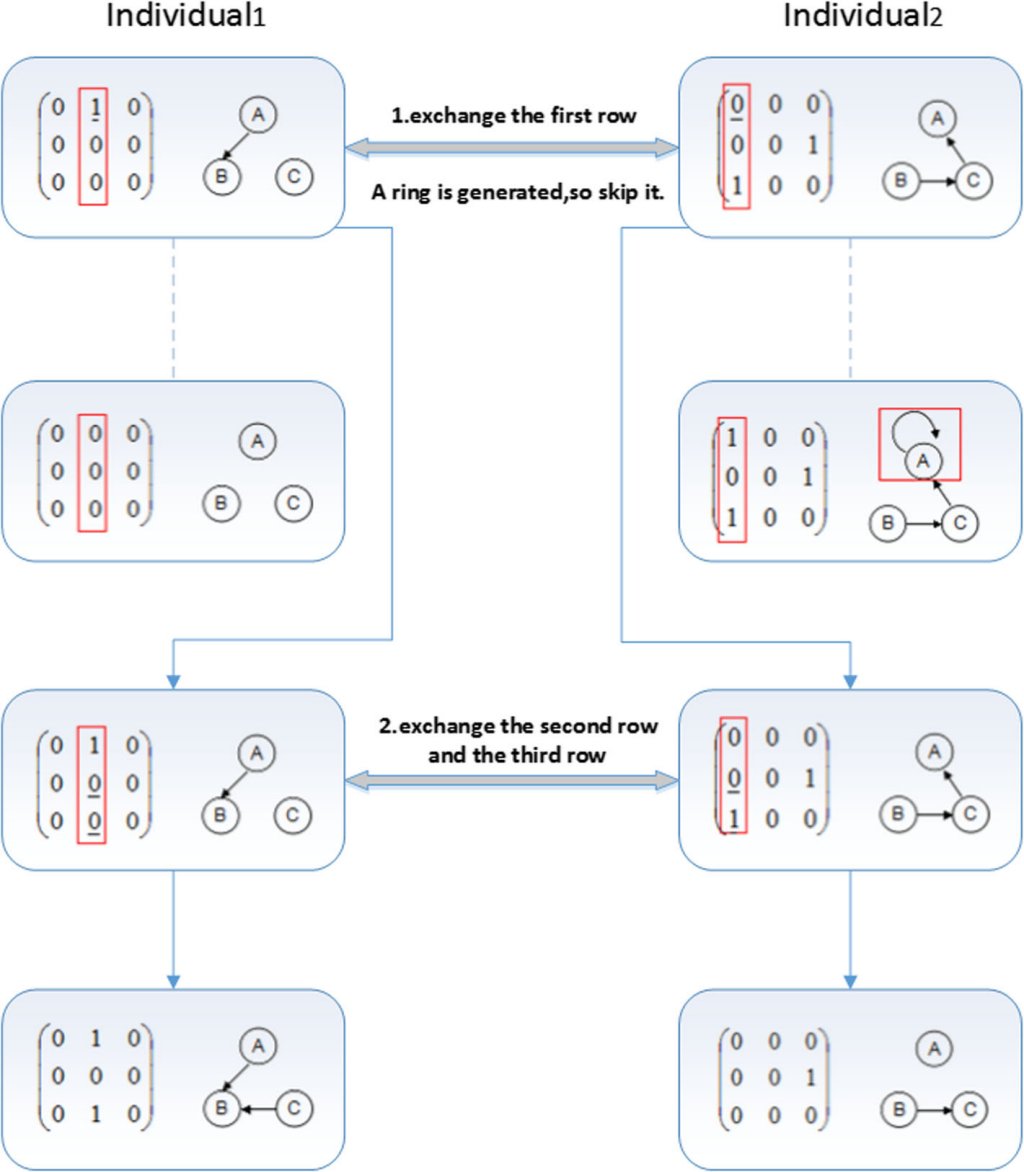
Process of avoid ring structure generation

If it will generate a ring structure when to exchange a row in the randomly chosen column in the process of crossover operation, the algorithm will skip that row. Then it judges the next row until all the two columns are exchanged. In Fig. 9, the crossover operation randomly selects two columns of *Individual*_*1*_ and of *Individual*_*2*_. Take a column pair for example, the second column is chosen in *Individual*_*1*_, and the first column is chosen in *Individual*_*2*_, as illustrated in the red mark of Fig. 9. The crossover operation is executed in the following two steps:

1. Exchange the first row in the chosen columns of two individuals. If the first row with the value of 1 in the second column of *Individual*_*1*_ is exchanged with the first row with the value of 0 in the first column of *Individual*_*2*_, we can see a ring structure in generated in *Individual*_*2*_, as illustrated in the red mark of Fig. 9. Then the crossover operation will skip the first row and do not exchange that row, then do the exchanging from the second row.
2. Exchange the second row and the third row, we can see no ring structure will be generated. The crossover operation will not skip these rows, and the final two individuals can be obtained after the crossover operation.

##### The general crossover operator

**Fig. 10:**
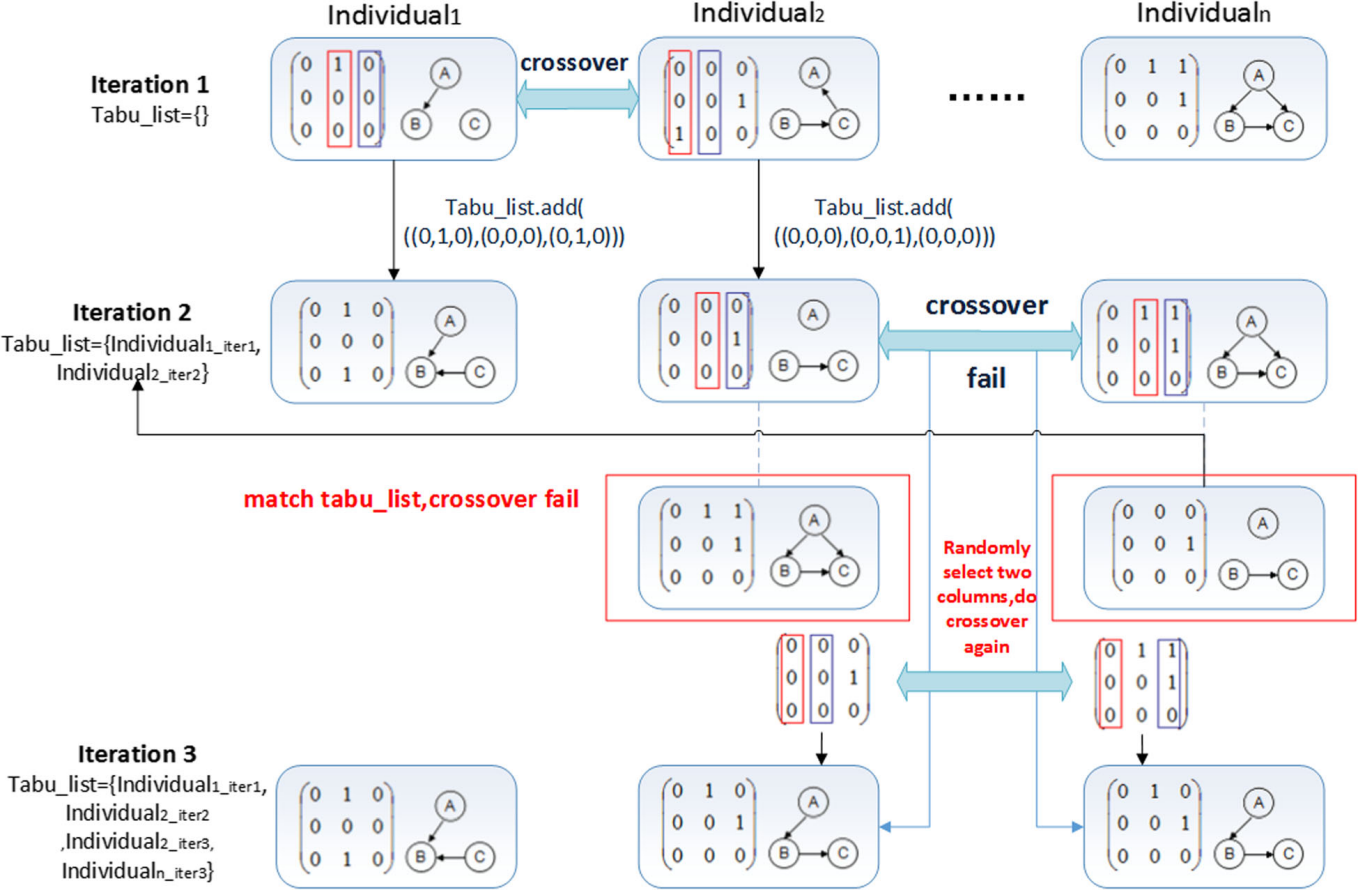
Process of crossover tabu operator

In the different offspring of particular population, crossover operation may produce the same offspring. This will cause the partial similarity of chromosomes in the population, and lead to search stagnant and prone to premature phenomenon. In Fig. 10, *Individual*_*1*_ and *Individual*_*2*_ are randomly chosen to do the crossover operation in iteration 1. Then we get two new offspring shown in iteration 2. In iteration 2, *Individual*_*2*_ and *Individual*_*n*_ are randomly chosen to do the crossover operation. In this way, two offspring are identical with the parents, as illustrated in the red mark. This crossover operation does not produce new offspring.

##### The crossover tabu operator

In order to solve the search stagnant and premature phenomenon that generated by general crossover operators, we use the memory function of tabu search method into the crossover operation of genetic algorithm. We compare the generated new offspring individual with the individuals in the tabu list one by one after each crossover operation, as illustrated in Fig. 6. If the new offspring individual does not belong to the tabu list, the algorithm will enter into the next generation and the new individual will be stored into the tabu list. The crossover operation will be carried out repeatly until the offspring are not belonging to the tabu list, as illustrated in Fig. 10. The detailed procedure is elucidated in Algorithm 1. 
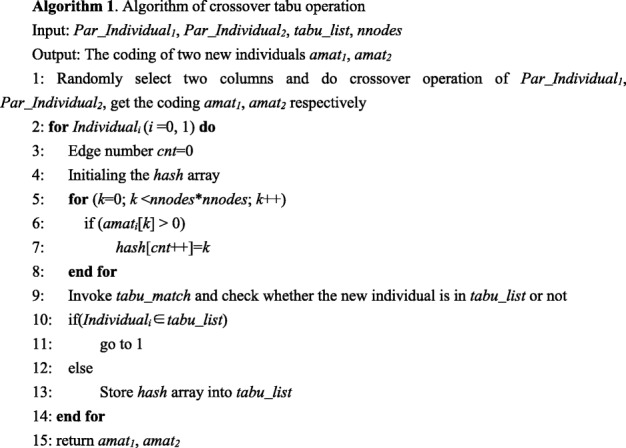


In Algorithm 1, *Par_Individual*_*1*_, *Par_Individual*_*2*_ are two parent individuals. *tabu_list* denotes the tabu table, and *nnodes* denotes the node number. In the output, *amat*_*1*_ and *amat*_*2*_ represent the coding of two new individuals.

#### Mutation

The mutation operator first selects an individual in the population randomly. The selected individual randomly changes the structure with a certain mutation probability *P*_*m*_. It is beneficial to increase the diversity of the population. The algorithm uses tabu mutation operator to select the mutation that has better variation fitness value, and it also ensures that a ring is not generated.

##### The general mutation operator

**Fig. 11:**
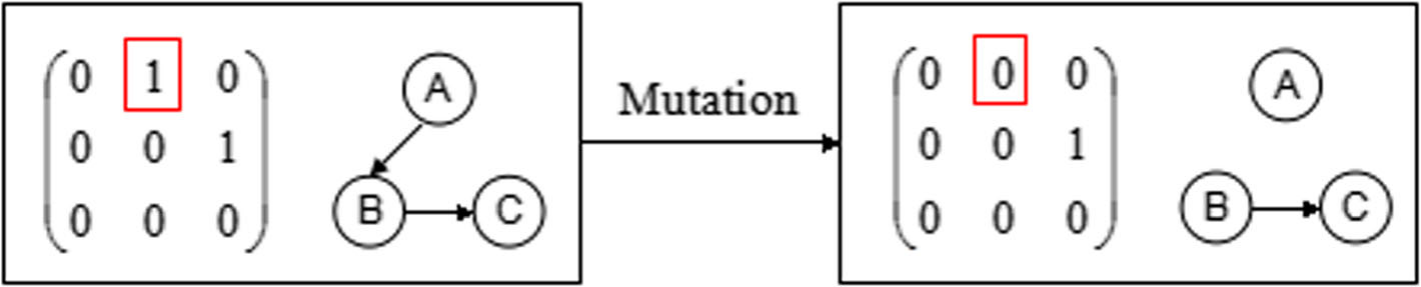
The general mutation operator

If the random probability is less than the mutation probability, it randomly selects a locus to perform mutation, as shown in Fig. 11. If the individual fitness is improved after the mutation and no ring structure is generated, then accept this individual and store it into the population.

##### The tabu mutation operator

The general mutation operator has strong randomness and may damage individuals with high fitness value. In order to solve this problem, we use the memory function of tabu list and propose a tabu mutation operator. This operator invokes evaluation function to determine the operation strategy. The new generated individual will be stored into the tabu list when the variation produces an inferior solution and improves the fitness value. The tabu mutation operator can avoid roundabout searches, and its climbing ability is better than the general mutation operator. The concrete process is illustrated in Algorithm 2. 
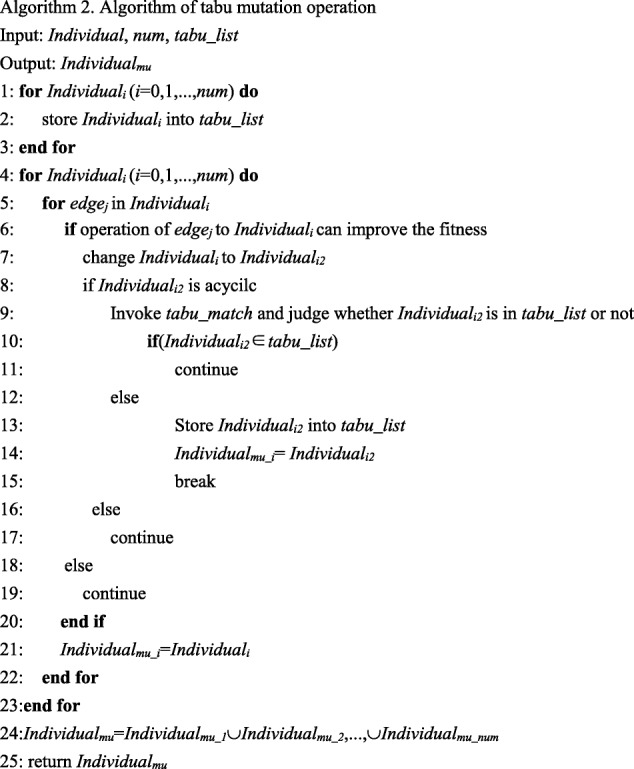


In Algorithm 2, *Individual* represents the current population, *num* represents the size of current population and *tabu_list* denotes the tabu table. *Individual*_*mu*_ represents the new individual generated by tabu mutation operation.

#### Fitness value evaluation

The fitness function is a standard to judge the quality of an individual or a solution. It determines which outstanding individuals are retained and which poorer individuals are eliminated. The genetic algorithm is an evolutionary search mechanism based on the fitness function. In this work, the fitness value evaluates the quality of the Bayesian network, and thus to guide the search strategy. In our method, the fitness value can be calculated using the *BN* scoring function. There are several kinds of BN scoring function, such as Bayesian Information Criterion (*BIC*), Akaike Information Criterion (*AIC*), Equivalent Dirichlet Posterior Density (*BDe*), *K2*, Log-likelihood, etc. To prevent over-fitting, we use the *BIC* scoring function to control the model complexity [45]. In addition, our experiment results demonstrate that the *BIC* scoring function has better learning effect.

In the case of given prior knowledge and sample data, Bayesian Information Criterion (*BIC*) selects the Bayesian network structure with the largest posterior probability. Supposing *D* represents the sample data, *G* represents the Bayesian network structure, we can get Eq.(10) using Bayesian formula. In the equation, *P*(*G*) represents the priori knowledge of network structure.

10 $$ P\left(G|D\right)=P\left(D|G\right)P(G)/P(D) $$

Using *θ*_*G*_ to denote the parameters of the network structure, we can get Eq.(11) through the marginal integration scheme.

11 $$ P\left(D|G\right)=\int P\left(D|G,{\theta}_G\right)P\left({\theta}_G|G\right)d{\theta}_G $$

The *BIC* scoring function is shown in Eq.(12).

12 $$ BIC\left(G|D\right)=\sum \limits_{i=1}^n\sum \limits_{j=1}^{q_i}\sum \limits_{k=1}^{r_i}{m}_{ij k}\lg \frac{m_{ij k}}{m_{ij\ast }}-\sum \limits_{i=1}^n\frac{q_i\left({r}_i-1\right)}{2}\lg m $$

In the equation, *m* represents the total number of samples. *n* represents the number of variables. *r*_*i*_ represents the number of values for the *i*th variable. *q*_*i*_ represents the combinations number of the parent about the *i*th variable. *m*_*ijk*_ represents the sample number of *i*th variable takes the *k*th value, and its parent nodes take the *j*th combination.

#### The end judgement

When the fitness value of an optimal individual reaches a given threshold, achieving the maximum number of iterations, or the fitness value of the optimal individual and the population no longer increases after *k* generations, then end up the algorithm.

## Additional file

Additional file 1: Epistatic interactions of AMD. (XLSX 35 kb)

## Data Availability

All data generated or analyzed during this study are included in this published article (and the additional information files). The code for this work is available at http://122.205.95.139/Epi-GTBN/.

## Abbreviations

AIC: Akaike Information Criterion
AMD: Real age-related macular degeneration
BDe: Equivalent Dirichlet Posterior Density
BEAM: Bayesian epistasis association mapping
BIC: Bayesian Information Criterion
BN: Bayesian network
BOOST: Boolean operation-based screening
Epi-GTBN: Epistasis mining based on genetic tabu algorithm and Bayesian network
GWAS: Genome-wide association study
MAF: Minimum allele frequency
MDR: Multifactor-dimensionality reduction
SNP: Single nucleotide polymorphism
